# Microbial strategies for lead remediation in agricultural soils and wastewater: mechanisms, applications, and future directions

**DOI:** 10.3389/fmicb.2024.1434921

**Published:** 2024-09-11

**Authors:** Isma Gul, Muhammad Adil, Fenglin Lv, Tingting Li, Yi Chen, Heli Lu, Muhammad Irfan Ahamad, Siqi Lu, Wanfu Feng

**Affiliations:** ^1^College of Geography and Environmental Science/Key Research Institute of Yellow River Civilization and Sustainable Development and Collaborative Innovation Center on Yellow River Civilization of Henan Province, Henan University, Kaifeng, China; ^2^Key Laboratory of Geospatial Technology for the Middle and Lower Yellow River Regions (Henan University), Ministry of Education/National Demonstration Center for Environment and Planning, Henan University, Kaifeng, China; ^3^Henan Dabieshan National Field Observation and Research Station of Forest Ecosystem, Zhengzhou, China; ^4^Laboratory of Climate Change Mitigation and Carbon Neutrality, Henan University, Zhengzhou, China; ^5^Xinyang Academy of Ecological Research, Xinyang, China; ^6^Henan Key Laboratory of Earth System Observation and Modeling, Henan University, Kaifeng, China; ^7^Department of Geography, Sustainability, Community, and Urban Studies, University of Connecticut, Storrs, CT, United States; ^8^The Forest Science Research Institute of Xinyang, Xinyang, Henan, China; ^9^Henan Jigongshan Forest Ecosystem National Observation and Research Station, Xinyang, Henan, China

**Keywords:** lead pollution, environmental toxicity, detoxification mechanisms, microbial and molecular remediation, environmental restoration

## Abstract

High lead (Pb) levels in agricultural soil and wastewater threaten ecosystems and organism health. Microbial remediation is a cost-effective, efficient, and eco-friendly alternative to traditional physical or chemical methods for Pb remediation. Previous research indicates that micro-organisms employ various strategies to combat Pb pollution, including biosorption, bioprecipitation, biomineralization, and bioaccumulation. This study delves into recent advancements in Pb-remediation techniques utilizing bacteria, fungi, and microalgae, elucidating their detoxification pathways and the factors that influence Pb removal through specific case studies. It investigates how bacteria immobilize Pb by generating nanoparticles that convert dissolved lead (Pb-II) into less harmful forms to mitigate its adverse impacts. Furthermore, the current review explores the molecular-level mechanisms and genetic engineering techniques through which microbes develop resistance to Pb. We outline the challenges and potential avenues for research in microbial remediation of Pb-polluted habitats, exploring the interplay between Pb and micro-organisms and their potential in Pb removal.

## 1 Introduction

**Figure 1:**
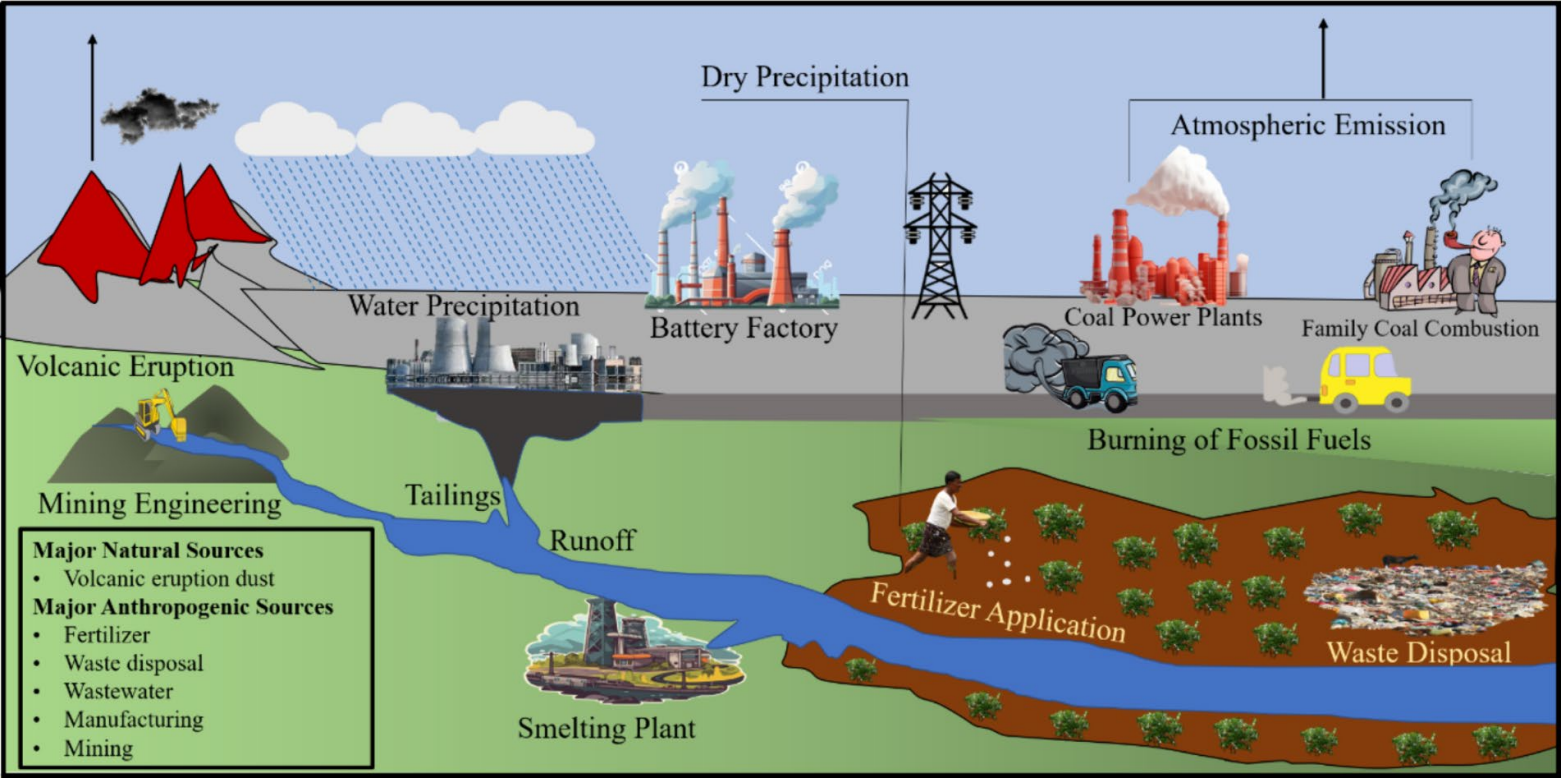
Presents the different sources of Pb pollution in the ecosystem [Modified from Figures in Hou et al. (2020) and Shan et al. (2023)].

Higher concentrations of heavy metals, the predominant contaminants in the environment, pose a significant hazard to soil and water due to their heightened toxicity levels (Ahamad et al., 2024; Razzaq et al., 2024; Zhao et al., 2024). Lead (Pb) has attracted considerable research attention worldwide due to its high toxicity, persistence, and accessibility (Futsaeter and Wilson, 2013; Raza Altaf et al., 2021; Shan et al., 2023). Hou et al. (2020) emphasized a substantial 232% rise in worldwide Pb production in the last five decades, reaching 11.3 Mt. annually due to industrial growth. The Pb contamination globally originates from natural and human-induced sources. Volcanic eruptions release natural Pb sources through dust emissions. Still, human activities, including mining, waste disposal, chemical plants, and fertilizer usage, have recently been the main causes of Pb pollution (Kushwaha et al., 2018). Different sources of Pb pollution in ecosystems are shown in Figure 1.

The progressive movement of elevated amounts of Pb contamination from the atmosphere, ground, and water sources into the food web and ultimately into body parts of humans presents heightened dangers. Previous research has shown that Pb poisoning can result in anemia, developmental issues, neurological disorders, and deaths in different animals (Wani et al., 2015) Similarly, Mitra et al. (2021) discovered that children in almost four million families globally are subjected to increased Pb levels, affecting their development and well-being. Pb contamination is a crucial issue in the worldwide environmental preservation system. Rahman and Singh (2020) classified methods for addressing Pb pollution into three main categories: physical, chemical, and biological treatments. Conventional physicochemical methods to remove Pb ions, such as chemical precipitation, ion exchange, membrane processing, and adsorption, encounter challenges due to high costs and inadequate Pb ion elimination (Dhankhar and Hooda, 2011).

**Figure 2:**
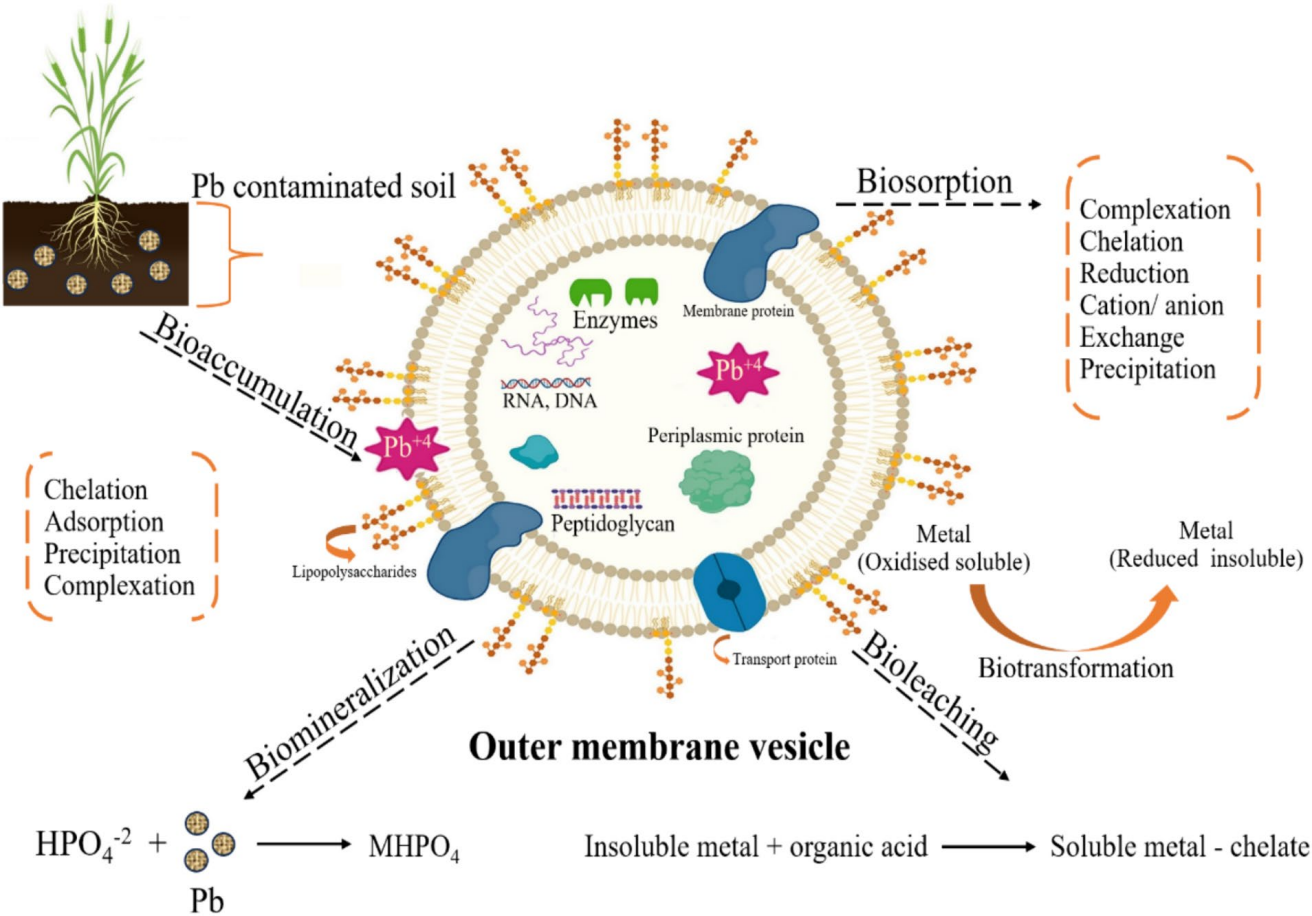
Different bioremediation methods, such as bioaccumulation, bioaccumulation, biomineralization, bioleaching, biotransformation, and biosorption, performed by the microbial system to remove or transform toxic Pb from contaminated sites [Modified from Figure in Joshi et al. (2023)].

**Figure 3:**
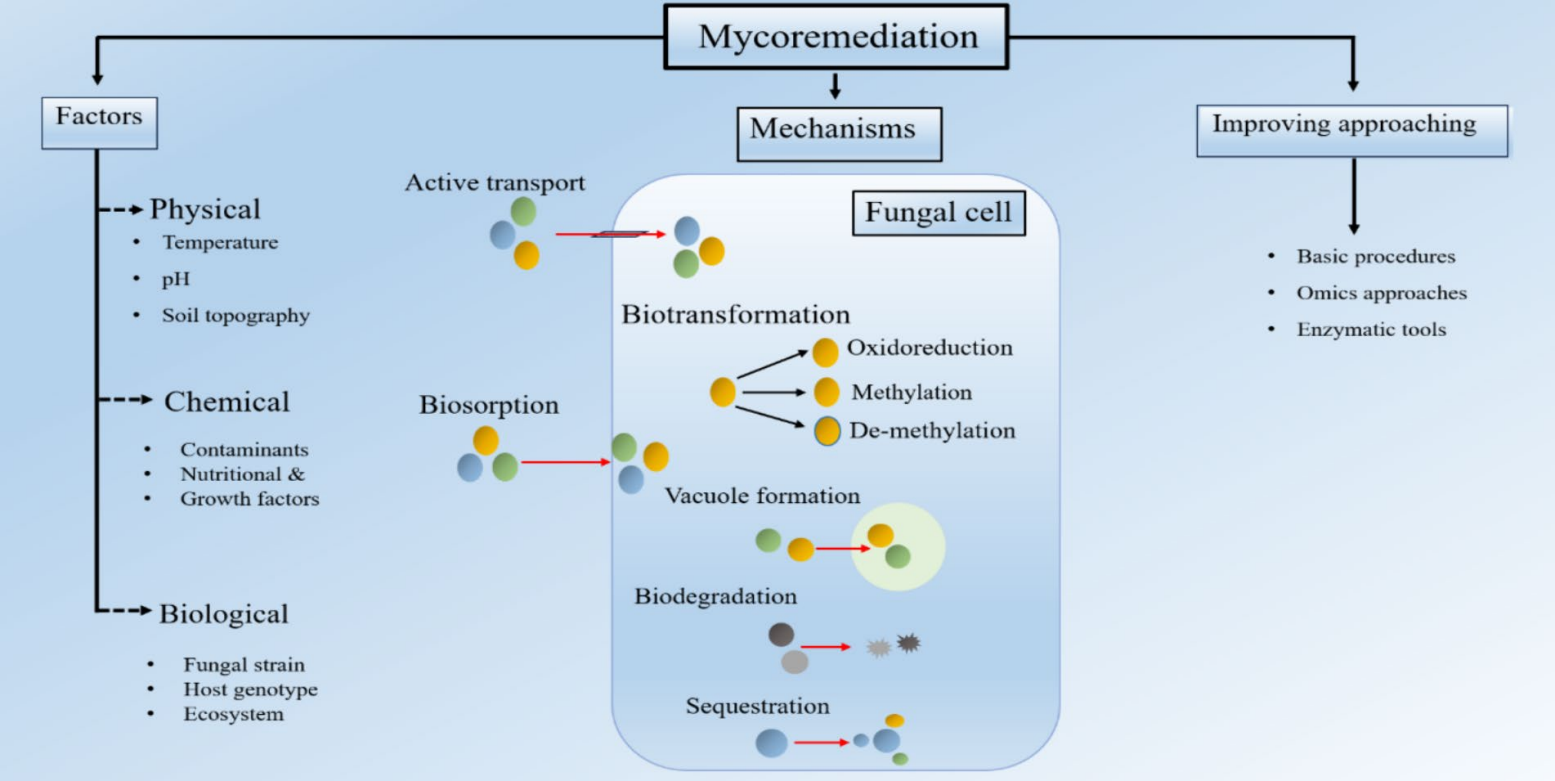
Fungi-assisted remediation of HM involves several mechanisms, such as biosorption, biodegradation, biotransformation, and sequestration [Modified from Figure in Kumar et al. (2021)].

**Figure 4:**
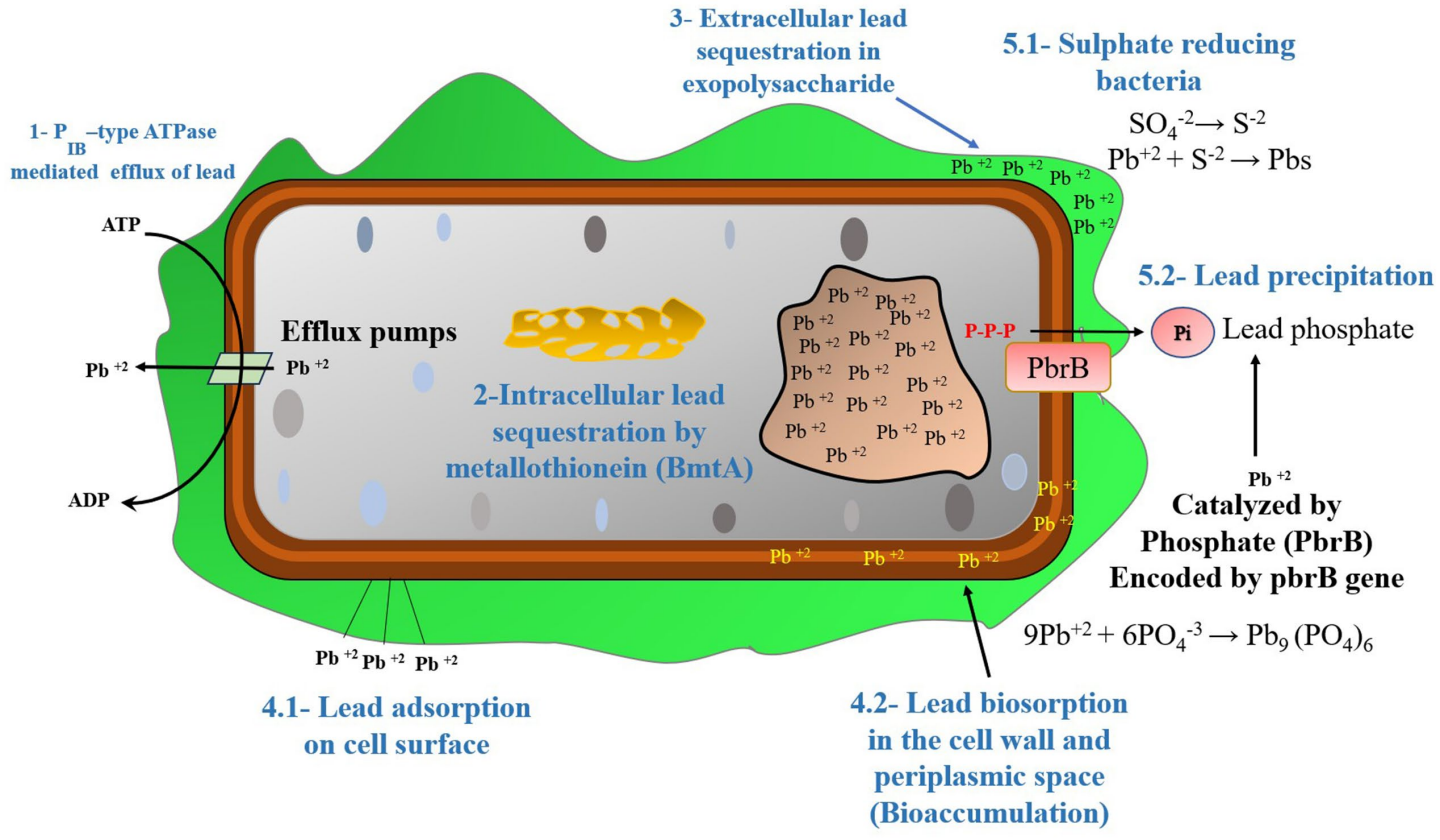
The Pb resistant mechanisms operational in bacteria, (1) PIB-type ATPase mediated efflux of Pb, (2) Pb sequestration by metallothionein (BmtA), (3) Pb sequestration in exopolysaccharide, (4.1) Cell surface adsorption of Pb, (4.2) Biosorption of Pb in the cell wall and periplasmic space (bioaccumulation), (5.1) Pb precipitation by sulfate-reducing bacteria, (5.2) Pb precipitation catalyzed by Phosphatase enzyme (PbrB) [Modified from Figure in Ashkan (2023)].

Microbial remediation is a simple, cost-effective, and efficient procedure compared to other alternatives (Adil, 2021; Raza Altaf et al., 2021; Wang et al., 2024). It entails adjusting environmental factors to stimulate the proliferation of micro-organisms and remove impurities. Critical techniques for eliminating Pb include biomineralization, bioprecipitation, biosorption, bioaccumulation, and efflux mechanisms, which convert soluble Pb ions into insoluble states (Figures 2–4). Studies have demonstrated that bacteria such as *Azotobacter chroococcum*, *Paenibacillus jamilae,* and fungus like *Aspergillus niger* are efficient in eliminating Pb from the environment (Xu et al., 2021; Shan et al., 2023). Progress in microbial technology has resulted in the creation of vigorous bacteria and the identification of associated genes, which may improve the effectiveness of removal procedures (Morillo Pérez et al., 2008).

This review suggests information about micro-organisms and their use in environmental cleanup, explicitly focusing on different types of bacteria, fungi, and microalgae recognized for their effectiveness in removing Pb. The study investigates the impact of environmental elements on the effectiveness of remediation, assesses procedures, and appraises the appropriateness of microbial-based techniques for Pb-contaminated locations. The article also addresses the obstacles and possibilities for extensive adoption.

## 2 Micro-organisms assisted remediation of lead-II

### 2.1 Bacteria assisted remediation

Bacteria can thrive in diverse environmental conditions, making them the most prevalent microbes on earth (Shan et al., 2023). Their varied origins, rapid growth, robust durability, and notable efficiency contribute to their extensive use in eliminating HMs. As shown in Table 1, recent global scientific studies have focused on finding native bacterial species that can eliminate the hazardous impacts of Pb. These include *Paenibacillus jamilae, Azotobacter chroococcum,* and *Sporosarcina pasteurii*. Bacterial assembly and adsorption are the primary techniques for eliminating Pb-II from the environment. Figure 2 shows the different bioremediation methods, such as bioaccumulation, bioaccumulation, biomineralization, bioleaching, biotransformation, and biosorption, performed by the microbial system to remove or transform toxic Pb from contaminated sites.

**Table 1:** Microbes-mediated removal of Pb with different environmental factors.

Micro-organism	Species	Temp (°C)	Optimal pH	Initial Pb concentration (mg L^−1^)	Pb removal rate	Removal mechanisms	References
Bacteria	*Pseudomonas stutzeri*	30	7	103.5	> 99.0%	Biosorption; bioaccumulation; bioprecipitation (calcium carbonate crystals containing Pb)	Wang et al. (2015)
	*Enterobacter cloacae* KJ-46	30	7	7.2	68.1%	Biosorption; bioprecipitation (PbCO_3_)	Kang et al. (2015)
	*Serratia marcescens*	28	7	100	97.57%	Biosorption; bioprecipitation (Pb_5_(PO4)_3_Cl)	Zhu et al. (2019)
	*Alishewanella* sp. WH16-1	37	6	100	84.13%	Biosorption; bioprecipitation (PbS)	Zhou et al. (2016)
	*Microbacterium oxydans* CM3	30	7.59	400	58.0%	Biosorption; bio accumulation; Bioprecipitation) (Pb_3_(PO_4_)_2_	Dabir et al. (2019)
	*Pseudomonas* sp.	20	7	50	> 90.0%	Biosorption	Gallardo-Rodríguez et al. (2019)
	*Bacillus subtilis* X3	37	4	300	192.05 mg g^−1^	Biosorption, biomineralization (Pb_5_(PO_4_)_3_OH, Pb_10_(PO_4_)_6_(OH)_2_ and Pb_5_(PO_4_)_3_Cl)	Qiao et al. (2019)
	*Bacillus cereus*	30	5	100	75.6%	Biosorption; complexing	Murthy et al. (2011)
Fungi	*Penicillium polonicum*	30	5	828.8	90.3%	Biosorption; bioprecipitation (PbC_2_O_4_, Pb_5_(PO_4_)_3_Cl)	Xu et al. (2020)
	*Aspergillus tubingensis*	30	5	828.8	> 90.0%	Biosorption; bio accumulation; Bioprecipitation (PbS, PbCO_3_, Pb_2_O_3.333_)	Shan et al. (2022)
	*Aspergillus niger*	30	5	828.8	97.0%	Biosorption; Bioprecipitation (PbC_2_O_4_)	Ding et al. (2019), Ding et al. (2019)
	*Trichoderma asperellum*	30	7	250	18.71%	Biosorption	Sun et al. (2018)
	*Rhizopus oryzae*	30	7	450	33.76%	Biosorption	Sun et al. (2018)
	*Mucor irregularis*	30	7	100	17.37%	Biosorption	Sun et al. (2018)
Microalgae	*Phormidium* sp.	25	5	10	2.305 mg g^−1^	Biosorption; Bio-accumulation	
	*Oscillatorialaetevirens*	25	5	60	20.36 mg g^−1^	Biosorption	
	*Pseudochlorococcu typicum*	20	7	10	4.49 mg g^−1^	Biosorption; Bio-accumulation	Shanab et al. (2012)
	*Spirulina (Arthrospira platensis)*	25	7	100	188 mg g^−1^	Bioaccumulation	Arunakumara et al. (2008)
	*Synechocystis* sp.	25	7	8	155.63 mg g^−1^	Bioaccumulation	Arunakumara et al. (2007)
	*Chlorella vulgaris* CCAP211/11B (Non-living)	28	7	600	39 mg g^−1^	Biosorption	Kumar et al. (2015)
	*Scenedesmus acutusI* FRPD1020 (Non-living)	28	7	600	90 mg g^−1^	Biosorption	Kumar et al. (2015)
	*Arthrospira* (Spirulina) *platensis* (Non-living)	25	5–5.5	About 621	102.56 mg g^−1^	Biosorption	Ferreira et al. (2011)
	*Aulosira fertilis-sima* (Non-living)	25	5	200	31.12 mg g^−1^	Biosorption	Singh et al. (2007)
	*Calothrix parietina* TISTR 8093	28	7	600	45 mg g^−1^	Biosorption	Kumar et al. (2015)
	*Chlamydomonas reinhardtii* (Caalginate immobilized)	25	6	100	230.7 mg g^−1^	Biosorption	Bayramoğlu et al. (2006)

Moreover, both living and dead bacterial biomass have significant capacities to absorb Pb-II, as Li et al. (2017) highlighted the exceptional biosorption abilities of *Pseudomonas* sp. -I3, a psychrotrophic bacterium tolerant to Pb-II. Similarly, the bacteria exhibited substantial Pb biosorption rates of 49.48 mg g^−1^ with living biomass and 42.37 mg g^−1^ with dead biomass. Bacterial cell walls include essential functional groups such as phosphate, carboxyl, sulfate, and amino groups that are important for Pb-II adsorption. Different types of bacteria have metal-binding spots on their cell walls and peptidoglycan such as the binding sites of Pb on the cell wall of Gram-positive bacteria and Gram-negative bacteria differ. In the cell wall of Gram-positive bacteria, the carboxyl group of peptidoglycans is the primary binding site for Pb, whereas in Gram-negative bacteria, the phosphate group plays a significant role. In addition, *Pseudomonas aeruginosa* PU21 has several negatively charged groups on its surface, which enhances its efficacy in extracting Cd, Pb, and Co from wastewater that is polluted with these metals (Shan et al., 2023).

Micro-organisms secrete extracellular polymeric substances, also known as EPS in a laboratory, mainly composed of nucleic acids, proteins, lipids, polysaccharides, and humic chemicals (Shan et al., 2023). EPS is crucial for heavy metal adsorption and the production of biofilms (Czaczyk and Myszka, 2007). Bacteria synthesize EPS to withstand environmental stress, exhibiting a high capacity to sequester metals inside the EPS framework. Moreover, the EPS has many ionizable functional groups that can bind metals better (Flemming and Wingender, 2010). Spectroscopic research has demonstrated that Pb-II has a higher affinity for phosphoryl groups in EPS produced by bacteria such as *Shewanella oneidensis* strain MR-1 (Ha et al., 2010). Molecular size and the presence of proteins, polysaccharides, and lipids influence the adsorption capacity of EPS (Comte et al., 2006). Chemicals like amino acids, sulfate esters, and high-nitrogen pyruvates help metals and ligands bind together (Loaëc et al., 1997).

### 2.2 Fungi assisted remediation

Fungi possess exceptional resistance to high amounts of heavy metals compared to bacteria and are very efficient at reducing these toxic substances in the environment. Fungi have more functional groups that can bind Pb-II and sequester it more effectively because their cell walls can make up to 30% of their dry mass (Dhankhar and Hooda, 2011). Similarly, Dhankhar and Hooda (2011) have demonstrated the performance benefits of an extensive fungi culture and short-cycle multiplication. In addition to this, many fungal biosorbents have non-pathogenic characteristics, making them ideal for engineering purposes. Shan et al. (2022) found that some fungi, like *Cunninghamella echinulate, Penicillium polonicum,* and *Aspergillus tubingensis*, absorb Pb-II well. Moreover, *Aspergillus niger* is a commonly used biosorbent for removing Pb-II.

Xu et al. (2021) exhibited that living and modified (high-temperature, freeze-dried, alkali treatment) *Aspergillus niger* has a great capacity to remove Pb(II) present in aqueous solution, and the rates of Pb(II) removal were 96.21, 8.76, 25.02, 15.05% under initial 828 mg L^−1^ Pb(II), respectively.

Using a response surface approach to improve the Pb-II adsorption by *Aspergillus niger* biomass intended changing the pH of the solution, the amount of Pb in it, and the dose of biomass, which led to a maximum adsorption capacity of 13.3 mg g^−1^ under pre-optimized circumstances (Amini et al., 2008).

Recent research has investigated using microbial composite methods for Pb cleanup. These methods involve mixing charcoal, sodium alginate, carbon fiber, and minerals with micro-organisms (Akar et al., 2013; Wang et al., 2022). Researchers have used fungal species like *Mucor plumbeus* and *Aspergillus niger* to create microbial composites that effectively remove Pb. Narayanan et al. (2021) demonstrated that the combination of *Aspergillus niger* and water hyacinth-made biochar can effectively adsorb and reduce pollutants. Ding et al. (2019) found that synthetic anatase may enhance the ability of *Aspergillus niger* to remove Pb(II) from aquatic environments. Although the final adsorption capacity did not show any noticeable fluctuation, the speed at which *Aspergillus niger* adsorbed Pb(II) increased by 204%. (Figure 3). The results highlight the possibility of using microbial composite techniques to enhance the removal of Pb pollutants from the atmosphere by leveraging the combined effects of micro-organisms and different materials.

### 2.3 Microalgae assisted remediation

Researchers worldwide are interested in microalgae because of their exceptional biological features, such as higher photosynthetic efficacy and strong growth in harsh atmospheres with high HMs levels, limited nutrients, and extreme temperatures (Shan et al., 2022). Because of their high tolerance and large number of surface binding sites, researchers increasingly use microalgae for the remediation of HMsin polluted areas. Priatni et al. (2018) stated that removing Pb through microalgae involves two steps. Initially, the external environment quickly absorbs Pb-II, gradually diffusing over the cell membrane and accumulating within the cell. The microalgae’s cell wall comprises laminaran, monomeric alcohols, deprotonated sulfate, and different functional groups, such as hydroxyl, amino, and carboxyl, which are the essential components for the Pb adsorption (Pradhan et al., 2019).

Several studies have shown that microalgae species like *Chlamydomonas reinhardtii*, *Aphanothece* sp., *Isochrysis galbana*, and *Chlorella sorokiniana* are effective in removing Pb-II from polluted environments (Table 1) (Hu et al., 2018; Keryanti and Mulyono, 2021; Li et al., 2021; Tan et al., 2022; Ye et al., 2022). Moreover, Zeraatkar et al. (2016) showed that variations in the cell wall composition and amount of various microalgae affect their ability to absorb Pb such as at the initial Pb-II level of 10 mg L^−1^, a removal efficiency of 92.2% was recorded.

## 3 Microbial Pb-II remediation mechanisms

Micro-organisms use the following remediation mechanisms to reduce the harmful effects of Pb (Figure 4).

### 3.1 Biosorption

Biosorption is vital for immobilizing Pb outside the cell to prevent its entry, which is achieved by many procedures, such as ion exchange, electrostatic interactions, and the cell wall binding of Pb-II (Chia et al., 2020; Shan et al., 2023). Pb-II biosorption often happens sequentially. The Pb-II biosorption rate first rises due to the abundance of accessible cell surface binding sites. However, the adsorption rate significantly decreases toward the end of the process as the binding sites fill up (Shan et al., 2023). Repulsive interactions between ions with similar charges may hinder the Pb-II adsorption on the cell surface (Sevak et al., 2021).

Researchers found *Pseudomonas aeruginosa,* a Pb-resistant strain, also known as 4EA, in polluted soil at an automobile battery disposal location in India. When researchers grew the cells in a solution comprising 166 mg L^−1^ of Pb-II, they noted a notable buildup of Pb on the cells’ surface (Naik and Dubey, 2011). The primary constituents of the cell wall consist of polysaccharides, chitin, and cellulose derivatives. These components include several functional groups that have the ability to effectively adsorb Pb(II). Several research have shown that carboxyl, hydroxyl, sulfhydryl, amine, and phosphonate groups have a role in the adsorption of Pb(II). These functional groups have the ability to form complexes with Pb(II) based on their ion exchange potential (Ha et al., 2010; Jin et al., 2016). The biosorption method for Pb(II) is not uniform but rather differs depending on the specific micro-organisms involved.

Mota et al. (2016) investigated the process by which *Cyanothece* sp. CCY 0110 absorbs Pb(II) ions; the results obtained from the research showed that the stretching vibrations of the hydroxyl and carboxyl groups on the cytoderm of the cell made it better at absorbing Pb-II onto its surface. Furthermore, several adsorption tests have conclusively shown that both living and non-living microbes can adsorb Pb(II). This is because high temperatures during the inactivation process remove some functional groups from dead biomass, making living biomass better at adsorbing Pb-II (Rahman et al., 2019). However, under some circumstances, dead biological material can adsorb a greater amount of metal ions than live organic matter due to pH and temperature control (Srinath et al., 2002). -The surfaces of microbes can adsorb Pb-II through covalent bonding, or non-covalent interactions. An adsorption isotherm, which demonstrates experimental behavior and allows for adsorption process prediction, assesses the biosorbent’s ability to adsorb Pb-II. Following the Freundlich model, the dissolved lead (Pb-II) sticks to different surfaces by multilayer adsorption.

Moreover, Guan et al. (2005) discovered that *Sphaerotilus natans* can take in all Pb-II at levels below 20 mg L^−1^. They also found that the process of absorption follows the Freundlich isotherm model. When there is minimal contact force between Pb-II molecules and microbial surfaces, the adsorption process adheres to the Langmuir model. Bacillus strain MRS-2 (ATCC 55674) tended toward the Langmuir isotherm model while adsorbing Pb-II, indicating a monolayer adsorption process (Hoyle-Gardner et al., 2021).

Fungus like *Saccharomyces cerevisiae* has shown the ability to capture and retain 65–79 percent of Pb and Cd from soil that is polluted (Lee et al., 2001) The biosorption process involves the utilization of fungal cell walls, which consist of chitin, proteins, glucans, lipids, pigments, and polysaccharides. These cell walls possess functional groups such as hydroxyl, carboxyl, amino, sulphate, or phosphate, and the process is facilitated by interactions such as adsorption, ion exchange, and complexation (Ojuederie and Babalola, 2017). Wood-decaying species, such as white-and brown-rot fungi, as well as mushrooms and other fungi, are used in mycoremediation due to their capacity to absorb heavy metals in their fruiting bodies (Jeyakumar et al., 2023).

The biosorption was first noticed in several microalgae during the early 1970s, when radioactive substances and heavy metals released from a nuclear reactor were accumulated in microalgae (Abdelfattah et al., 2023). The cell wall of microalgae is directly accountable for biosorption, and its chemical composition plays a crucial part in the process. Furthermore, microalgal surfaces possess holes, and the presence of surface charge facilitates biosorption. The cell wall of microalgae contains many chemical groups, including carboxyl, hydroxyl, and sulfate. These groups serve as binding sites and also act as ion exchangers, facilitating the complexation of metal ions and the adsorption of organic compounds from contaminated water (Soto-Ramírez et al., 2021).

Moreover, the cell surface’s active binding sites have the capability to create complexes with certain contaminants found in water. This process triggers flocculation and leads to a decrease in the overall amount of dissolved and suspended solids (Al-Tohamy et al., 2022). The process of HMs ions biosorption by microalgae occurs via a two-step method. The process comprises two stages. The first stage is metabolism-independent and involves the quick and reversible binding of adsorbate onto active sites on the surface of microalgae. The second step is slower and involves positive intracellular diffusion, predominantly driven by the metabolic activity of microalgae (Abdelfattah et al., 2023).

### 3.2 Bioaccumulation

Bioaccumulation refers to the accumulation of elements or compounds by organisms from their environment as they grow, storing these substances in their bodies. Transporters and passive diffusion facilitate the transfer of Pb ions into microbial cells, which move from zones of high absorption to zones of low absorption. The dissolved lead (Pb-II) can penetrate the cell core and build up even after adhering to the cell surface (Arifiyanto et al., 2017). Pb ions can form associations with cytoplasmic molecules upon entering the cell or diffuse into vacuoles. Active microbes accumulate more Pb in their cells than inactive biomass due to proteins with vital biological roles. *Bacillus coagulans* R11 cells that were active could eliminate a lot of Pb up-to 17.53 mg g^−1^ of Pb-II in the best conditions (Xing et al., 2021).

According to Xing et al. (2018), bacteria in active growth accumulated more Pb within their cells than dormant spores, suggesting an active transport system for Pb-II absorption into the cell. Further, Liu and Yen (2016) studied the sulfate-reducing bacteria, specifically *Shewanella oneidensis,* in which the cells absorbed Pb-II by passive diffusion. The growth stage of micro-organisms influences the rate of Pb bioaccumulation, peaking during the logarithmic growth phase and then declining over time (Sizentsov et al., 2019). Biological components such as cysteine, cytosolic polyphosphates, sulfide, and glutathione can combine with Pb to protect against Pb-II exposure. Huang et al. (2016) suggested three conserved cysteines might interact with Pb-II in a trigonal-pyramidal coordination configuration. Similarly, Gadd and White (1993) discovered some proteins exhibiting distinct reactions to Pb ions, while Li et al. (2022) elucidated how cytoplasmic proteins, including thioredoxin (TXN) and formaldehyde-activated enzyme (GFA), assist in the interaction of Pb-II with glutathione within cells, offering protection against toxicity. Cells contain a significant amount of metallothioneins (MTs), characterized by their high sulfhydryl amount and low molecular weight. Metallothioneins can accumulate heavy metal ions in living organisms, assisting in their growth and chemical processes (Blindauer et al., 2002; Liu et al., 2003).

*Pseudomonas aeruginosa* strain WI-1 may accumulate Pb-II internally using the bmtA gene, which produces MTs, with a maximum capacity of 26.5 mg g^−1^ (Naik et al., 2012a). Similarly, *Salmonella choleraesuis* strain 4A was shown to have genomic DNA including MTs (SmtA), associated with Pb resistance, and capable of accumulating up to 19 mg g^−1^ (Naik et al., 2012b). Arifiyanto et al. (2017) observed the presence of microtubules in *Bacillus* sp. following exposure to Pb. These MTs helped make Pb complexes inside the cytoplasm.

Previous studies by Xu et al. (2014) and Zhang et al. (2016) confirm that certain fungi have the ability to gather significant quantities of heavy metals by binding them with glutathione (GSH) within their cells without causing any damage to the cell structure. However, the majority of research found in the literature about the removal of Pb(II) primarily concentrate on the choice of fungal strains, the optimization of environmental conditions, and the measurement of removal effectiveness. Only a small number of studies have examined the processes involved in the elimination of Pb(II). In addition, a smaller number of articles examined the processes of Pb(II) elimination specifically from the perspective of minerals containing Pb. *Penicillium polonicum*, a filamentous fungus, was obtained from the effluent of a lead-zinc mine located in Dexing City, Jiangxi Province, China (Xu et al., 2020). The fungus was confirmed to have the ability to tolerate Pb(II) concentrations of up to 12 mmol L^−1^ (2486.4 mg L^−1^) 13 and had a high efficiency in removing Pb (Yang et al., 2012).

Understanding the bioaccumulation mechanism is essential for effectively addressing Pb pollution in affected areas. Researching micro-organisms that can collect Pb can potentially improve Pb cleanup methods. Enhancing our understanding of Pbaccumulation and identifying micro-organisms with exceptional abilities will help us develop precise and efficient cleaning strategies. This field of study shows promise for mitigating the detrimental effects of Pbpoisoning on the environment and human health. A list of microbes reported to increase plant growth and productivity under Pb stress compared to control is shown in Table 2.

**Table 2:** A list of microbes reported to increase plant growth and productivity under Pb stress compared to control.

Microbial inoculants	Plant species tested	Effect on plants	References
*Pseudomonas putida* KNP9	*Vigna radiata*	Increase in root growth-20% Increase in shoot growth-19%	Tripathi et al. (2005)
*Burkholderia* sp. J62	*Zea mays*	Overall increase in plant growth and productivity-75%	Jiang et al. (2008)
	*Solanum lycopersicum*	Overall increase in plant growth and productivity-30–54%	
*Pseudomonas fluorescens*	*Brassica napus*	Overall increase in plant growth and productivity-21%	Sheng et al. (2008)
*Microbacterium* sp. G16		Overall increase in plant growth and productivity-35%	
*Bacillus* sp. SC2b	*Sedum plumbizincicola*	Overall increase in plant growth and productivity-42-46%	Ma et al. (2015)
*Funneliformis mosseae*	*Robinia pseudoacacia*	Increase in root growth-28%, Increase in shoot growth-43%	Yang et al. (2015)
*Rhizophagus intearadices*		Increase in root growth-38% Increase in shoot growth- 75%	
*Phialocephala fortinii*	*Clethra barbinervis*	Overall increase in plant growth and productivity- 376%	Yamaji et al. (2016)
*Rhizodermea veluwensis*		Overall increase in plant growth and productivity- 157%	
*Rhizoscyphus* sp.		Overall increase in plant growth and productivity-213%	
*Bradyrhizobium japonicum*	*Lactuca sativa*	Increase in root growth- 42%, Increase in shoot growth-28%	Seneviratne et al. (2016)
*Bacillus* sp.QX8 and QX13	*Solanum nigrum*	Overall increase in plant growth and productivity- 1.36 fold	He et al. (2020)
*Bacillus spizizenii* DSM (SN36)	*Spinaacia oleracea* L	Overall increase in plant growth and productivity- 170%	Desoky et al. (2020)
*Paenibacillus jamilae*		Overall increase in plant growth and productivity- 179%	
*Pseudomonas aeruginosa*		Overall increase in plant growth and productivity- 205%	
*Pseudomonas* spp.	*Anethum gravelens* L	Overall increase in plant growth and productivity- 117%	Rahbari et al. (2021)

Microalgae have the ability to gather various contaminants together with nutrients and microelements that are already present (Mustafa et al., 2021). Microalgae has the ability to adapt to their surroundings, enabling them to withstand low quantities of contaminants. In addition, microalgae have a high level of tolerance to various contaminants originating from residential, agricultural, and industrial sources, hence enhancing their potential to remediate these pollutants (Wu et al., 2012; Mojiri et al., 2020). In order to enhance the effectiveness of microalgae in bioremediation, it is essential to adjust the physicochemical conditions. This is because the pace and capacity of microalgae in the bioaccumulation process are dependent on these factors. Furthermore, the process of selecting microalgae species that can withstand high levels of pollutants is a successful approach to increase the ability and speed of bioaccumulation (Abdelfattah et al., 2023).

### 3.3 Bioprecipitation and biomineralization

Biosorption and bioaccumulation frequently occur alongside precipitation and mineral formation, which may postpone toxicity’s inception. Bioprecipitation and biomineralization are vital in decreasing Pb-II availability and aiding in environmental reuse (Figure 3). Micro-organisms can immobilize Pb-II by causing it to precipitate, a process known as intracellular or extracellular biomineralization (Figure 4).

#### 3.3.1 Micro-organisms engaging in extracellular biomineralization

Micro-organisms frequently excrete oxalic acid as an external metabolite. The solubility of oxalic acid reduces dramatically when it forms a chelating complex with Pb-II metal cations. Xu et al. (2020) showed that Pb-II notably boosts the release of oxalic acid by *Penicillium polonicum*. The response is due to the fungal stress reaction, which involves creating lead oxalate minerals outside the cell to reduce Pb toxicity. Ding et al. (2019) found that living cells of *Aspergillus niger* may trap Pb-II as lead oxalate on their cell wall.

According to Park et al. (2011), microorganism-induced phosphate precipitation is a cost-effective, viable, and ecologically friendly approach to address Pb pollution. Phosphate-solubilizing bacteria (PSB) are essential for breaking down phosphate using enzymes such as phytase or phosphatase, which helps increase phosphorus availability in soil. Scientists worldwide are interested in immobilizing Pb-II by using available phosphates to react with Pb, transforming it into less soluble forms such as lead phosphates (Wang et al., 2020). Although immobilizing Pb-II with phosphates may lead to a decrease in plant-accessible phosphorus, it remains a feasible method in agricultural areas when handled with caution. Farmers may reduce Pbhazards and preserve soil fertility and crop production by using accurate application techniques, utilizing alternate phosphorus sources, adopting effective crop management practices, and implementing constant monitoring. The effectiveness of the phosphorus amendment in lead-contaminated soil is contingent upon the soil type, as well as the characteristics and magnitude of the contamination. Thorough analysis should be conducted on the kind and rate of the P source, as well as the application management, for soil amendment (Miretzky and Fernandez-Cirelli, 2008).

The microbes release enzymes such as phosphatases to break down β-glycerol phosphate, releasing PO_4_^3−^ions that react with Pb-II to form a precipitate (Naik and Dubey, 2013). General anions such as fluoride (F^−^), chloride (Cl^−^), and bromide (Br^−^) can assist in PbII mineralization when PSB is present. Qiao et al. (2019) proposed that *Bacillus subtilis* X3 may convert Pb-II into Pb_5_(PO_4_)_3_OH and Pb_5_(PO_4_)_3_Cl by mineralization, such as Su et al. (2020) found Pb_5_(PO_4_)_3_OH on the *Rhodobacter sphaeroides* SC01 cell membrane. This is primarily because of the complex interaction between the phosphate group and Pb ions. Researchers have already found that microbial metabolites and organic chemicals can speed up the breakdown of pyromorphite, which lets the Pb out (Debela et al., 2010; Topolska et al., 2013). Thus, using PSB for Pb cleanup still presents a notable issue.

Moreover, micro-organisms containing urease enzymes may efficiently trap Pb-II together with PSB. They drive the process by catalyzing urea hydrolysis, increasing pH levels to between 8.0 and 9.1, and generating CO_3_^2−^ ions, which encourage the creation of calcium and lead carbonates (Shan et al., 2021). Stocks-Fischer et al. (1999) found that when conditions are alkaline, calcium ions in the calcium carbonate lattice may eventually be replaced by Pb ions. This can cause composite calcium-lead carbonate precipitates to form. Achal et al. (2012) were the first to introduce microbial carbonate precipitation procedures for treating Pb-contaminated soils. They showed that *Kocuria flava* efficiently traps Pb by generating lead oxide and carbonate. Furthermore, lead ions (Pb-II) can transform into calcite crystals when they touch a cell wall. The carbonate precipitation induced by Microbes for remediating Pb pollution is mostly experimental or restricted in scale due to restrictions linked to nutrition supply, calcium supplementation, urea availability, and microbial mobility.

#### 3.3.2 Micro-organisms engaging in intracellular biomineralization

Micro-organisms can precipitate Pb ions by generating extracellular metabolites. Additionally, the movement of Pb ions into cells might result in their immobilization via biological mechanisms. Xu et al. (2020) showed that *Penicillium polonicum* could transfer Pb from the external environment into the cytoplasm and then transform it into Pb(0) with the help of reductase enzymes. Sani et al. (2010) highlighted the importance of goethite and lead chloride treatment in producing compact lead/iron sulfide precipitates in the cytoplasm and periplasm of *Desulfovibrio desulfuricans* G20. Levinson et al. (1996) found that high concentrations of Pb(NO_3_)_2_ can lead to the movement of Pb-II into the cytoplasm of *Staphylococcus aureus*, leading to the creation of lead phosphate [Pb_3_(PO_4_)_2_] deposits.

*Providentia alcalifaciens* may immobilize the Pb strain 2EA and *Vibrio harveyi*, creating Pb_9_(PO4)_6_ precipitates inside the cells. Li et al. (2022) conducted a proteomics study that showed how enzymes triggered by thioredoxin and glutathione in the presence of formaldehyde may help create Pb-glutathione composite precipitates within cells. Shan et al. (2022) used Selected Area Electron Diffraction (SAED) patterns and Field Emission High-Resolution Transmission Electron Microscopy (FE-TEM) to study the formation of unique lead oxides (Pb_2_O_3.333_) in *Aspergillus tubingensis* cells grown in a solution with 828 mg L^−1^ of Pb-II. Their research exhibited that Pb-II was oxidized, leading to the creation of lead oxide precipitates inside *Aspergillus tubingensis* cells. The exact process of how Pb forms within cells is not well known because of the intricate interaction between minerals and bacteria.

Sayer et al. (1999) exhibited that an *Aspergillus niger* produced lead oxalate and lead oxalate dihydrate through microbial phosphate-solubilizing mechanisms during the transformation of pyromorphite [Pb_5_(PO_4_)_3_Cl]. This discovery marked the first recorded instance of the biogenesis of this mineral. Subsequent studies have revealed that numerous bacteria and fungi, when exposed to different environmental conditions, are capable of immobilizing Pb ions. They achieve this by converting inorganic phosphate sources, such as apatite minerals, or organic phosphate sources, such as phenolphthalein diphosphate, glycerophosphate, acephate, glycerol 2-phosphate, and phytic acid, into phosphate. This conversion process occurs in the presence of either phosphatase or phytase enzymes.

### 3.4 Efflux mechanisms

For optimal growth of micro-organisms, it is crucial to manage the concentration of harmful heavy metals within cells using efflux mechanisms (Nies, 1999). Various micro-organisms, especially those in polluted settings, exhibit heavy metal outflow (Yin et al., 2019). Multiple groups of membrane transporters facilitate exocytosis at the plasma membrane. The transporters can be classified into different groups, including the ATP-binding cassette (ABC), multidrug endosomal transporter (MET), and resistance-nodulation-cell division (RND) group. Resistance genes carried on plasmids primarily serve to control the activity of metal ion transporters.

Nevertheless, the particular micro-organism and heavy metal ions can impact the nature of this interaction. Specific transporter proteins hinder the excessive buildup of Pb-II in cellular structures. P-type ATPases, classified as transmembrane transport proteins, enable the transportation of tiny organic molecules and ions across cellular membranes (Coombs and Barkay, 2004).

**Figure 5:**
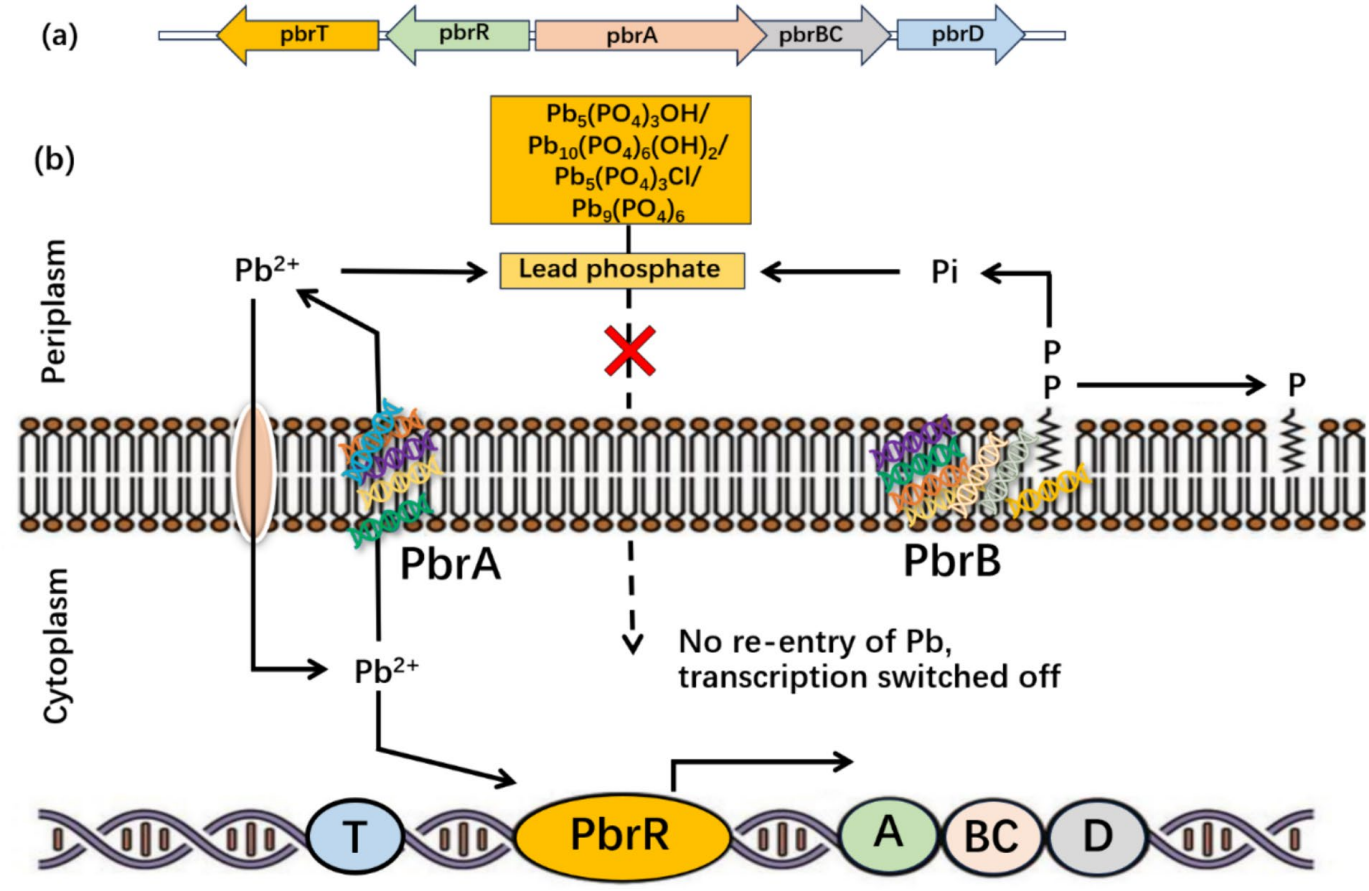
Bacterial response to Pb toxicity at the molecular level. **(A)** Pb-resistant genes pbrTRABCD in *Cupriavidus metallidurans* CH34 and **(B)** stimulation of Pb-resistant genes and generation of lead phosphate in bacteria [Modified from Figure in Hynninen (2010)].

According to Hynninen et al. (2009), the *Cupriavidus metallidurans* CH34 can tolerate Pb-II by removing it from the cytoplasm via P-type ATPase (Figure 5).

The zntA gene in *Escherichia coli* makes Pb-II-translocating ATPases, similar to the cadA gene in the pI258 plasmid from *Staphylococcus aureus*. Both genes are implicated in Pb-II translocation, as reported by Rensing et al. (1999). Furthermore, P-type ATPases transport HMs from the cytoplasm to the periplasm. An ion-proton exchanger called the CBA efflux pump moves metal ions out of the periplasm and into the extracellular space. Among the P-type ATPases, PIB-type ATPases are significant because they eliminate Pb-II, maintain homeostasis, and prevent Pb-II poisoning (Rensing et al., 1999). Similarly, Coombs and Barkay (2004) revealed that PIB-type ATPases facilitate Pb-II transport via the cell wall. Most sequenced archaea, bacteria, and eukaryote genomes have around one hundred genes that encode PIB-type ATPases. Moreover, Argüello et al. (2003) studied how heavy metals affect PIB-type ATPases in the thermophile *Archaeoglobus fulgidus*, focusing on the CopA enzyme. Researchers discovered that certain heavy metals could potentially trigger the CopA enzyme. The operon pbrUTRABCD in *Ralstonia metallidurans* was sequenced by Taghavi et al. (2009) to control Pb-II and lessen toxicity and hazardousness.

## 4 Factors affecting Pb-II elimination by micro-organisms

### 4.1 Effects of pH

The pH of the environment significantly influences the availability of Pb to micro-organisms, affecting microbial biomass and enzyme activity. Scientists who study how pH affects oxidation–reduction enzymes like phosphatase and urease have pointed out how important they are for microbes to keep Pb ions from moving. pH has a considerable impact on the precipitation of Pb (Shan et al., 2022). The cell wall’s functional groups strongly bind to H3O+ ions at low pH, generating repulsive interactions restricting the Pb-II adsorb and precipitating on the cell wall. The best pH range for removing Pb-II from microalgae-immobilized biomass is between 5 and 6. As the pH rises, Pb(OH)_2_ precipitates form. Decreased pH levels reduce the effectiveness of removing Pb-II because Pb ions and hydrogen ions compete for adsorption sites (Akhtar et al., 2004).

Moreover, *Bacillus subtilis* FZUL-33 has been shown to co-precipitate Pb-II when the pH is above 5.5, and the mineral’s shape is strongly influenced by pH (Lin et al., 2016). Li et al. (2013) found that *Sporosarcina pasteurii* strains speed up the biomineralization process of Pb. This process turns Pb into solid lead carbonate crystals when the pH level is between 8 and 9. pH affects the crystallization of Pb ions and also influences the production of humic and fulvic acid by bacteria. Pérez-Esteban et al. (2019) suggested a direct relationship between pH levels and the amounts of humic and fulvic acids in the environment. Humic chemicals can decrease the movement of Pb ions in the environment by forming complexes (Shan et al., 2022). To find the best pH to eliminate Pb-II, it’s essential to do a complete analysis of different micro-organisms, considering the amount of secretion, the level of ionization, the adsorption sites, and the surface charge of the adsorbent. The pH of the environment significantly influences the availability of Pb to micro-organisms, affecting microbial biomass and enzyme activity. Scientists who study how pH affects oxidation–reduction enzymes like phosphatase and urease have pointed out how important they are for microbes to keep Pb ions from moving. pH has a considerable impact on the precipitation of Pb (Shan et al., 2022).

### 4.2 Effects of temperature

The efficiency of removing Pb-II differs across various microbial species and is influenced by temperature changes. Temperature impacts microbial activity, including secretion content, biomass, and enzyme activities related to Pb-II binding. Temperature significantly influences the rate of growth of phosphate-mineralizing bacteria (PMB) (Qian and Zhan, 2016). The biomass of PMB reached its highest point at 30°C, leading to a substantial production of alkalinity material and phosphatase throughout the growth process. Zucconi et al. (2003) discovered that treating Paecilomyces lilacinus with high temperatures made living cells adsorb more Pb-II than dead cells. This suggests that high temperatures may stop enzymes that bind Pb from working. Micro-organisms showed an enhanced capacity to adsorb Pb-II within a certain temperature range as temperature increased (Bandowe et al., 2014).

Furthermore, extracellular polymeric substances (EPS) significantly impact Pb-II adsorption, and their formation is linked to temperature. EPS production by different bacteria takes place between −2°C and 42°C, with the amount of EPS generated being influenced by the ideal temperature for microbial development. Most microbes usually release more EPS at 25°C and 30°C (Takeda et al., 1991; Nichols et al., 2005; Zheng et al., 2008; Buthelezi et al., 2010). Choosing the best temperature for EPS production and microbial growth is crucial, considering the appropriate microbial species and environmental conditions. The efficiency of removing Pb-II differs across various microbial species and is influenced by temperature changes. Temperature impacts microbial activity, including secretion content, biomass, and enzyme activities related to Pb-II binding.

### 4.3 Biostimulation and bioaugmentation

Micro-organisms that can handle Pb are often found in places where Pb is present. These micro-organisms are very resistant to oxidation and have ways to get rid of Pb (Li et al., 2020). These native organisms are essential in the biogeochemical process of heavy metal remediation. Researchers have proven that biostimulation techniques, such as providing more nutrients, electron donors, or acceptors, enhance the resilience of micro-organisms in Pb polluted areas, leading to higher immobilization or transformation of Pb pollutants (Hou et al., 2020). Similarly, Huang et al. (2006) grew *Phanerochaete chrysosporium* in Pb-contaminated fields, which led to the breakdown of straw and the creation of humus, which may bind and trap Pb-II ions.

Moreover, biofertilizers stimulate the growth and development of native micro-organisms and increase the synthesis of organic matter that interacts with soil to create significant clusters of organic minerals and materials (Wang et al., 2019). Bioaugmentation, also known as *in-situ* bioremediation, introduces lab-grown bacteria capable of handling Pb in polluted areas (Yu et al., 2016). Current bioaugmentation approaches mainly include utilizing laboratory-cultivated microbial strains to create biofertilizers that enhance plant development. However, these laboratory-cultivated micro-organisms typically face challenges when competing with natural species.

Additional study is necessary to enhance the practicality and effectiveness of lab-grown microbes in immobilizing Pb in polluted areas. Due to the complex biological structures of contaminated areas and their adjacent surroundings, achieving the desired results with only one remediation method might be difficult. Integrating several approaches customized to the individual needs of real-time remediation is essential for maximizing the effectiveness of Pb-contamination cleanup.

## 5 Genetic-engineering techniques

Bioremediation research has turned to genetic engineering as a viable method because it enhances microbes’ resistance to metal stress, boosts the production of metal-binding proteins, and expands the storage capacity of metal. Genetic engineering methods usually entail inserting individual genes or operons and altering current gene sequences to create new strains with distinct metal-binding properties.

Four primary techniques are being considered in developing genetically engineered microbes for bioremediation (Jeyakumar et al., 2023). These techniques include: (1) using bio-affinity bioreporter sensors to sense chemicals, analyze end points, and reduce toxicity; (2) creating, monitoring, and controlling bioprocesses; (3) enhancing affinity and enzyme specificity; and (4) constructing and regulating routes. The choice of cellular factories is a crucial element that must be taken into account. Within the realm of fungus, bacteria, and algae strains, the bacterial system has been shown to possess significant promise due to its inherent characteristics, including a faster growth rate, confinement, and ease of genetic modification. However, cyanobacteria and microalgae contribute to sustainability and economic feasibility due to their photosynthetic activities and sophisticated metabolic pathways, similar to those found in the plant kingdom. The main function of biosorption and bioaccumulation is to remediate heavy metals. Efforts are being made to develop techniques that increase the adsorption of heavy metals on cell surfaces and boost the capacity to accumulate these metals by introducing porters (Diep et al., 2018). For instance, certain bacteria absorb and increase the expression of metal ion import systems such as channels, main active transporters, and secondary carriers to enhance the absorption of particular heavy metals.

Additionally, there is a strong focus on significantly reducing or treating heavy metals after their buildup in living organisms. Enzymes and proteins that are specially intended to decrease heavy metal complexes are added to various species to improve their ability to remediate these contaminants. Metal importers, which rely on enhanced diffusion processes mediated by channel proteins, were incorporated to enhance the absorption of arsenic and mercury ions (Diep et al., 2018). Researchers have shown increasing interest in cell surface engineering, specifically in expressing metal-specific peptides in the extracellular phase to improve adsorption and remediation processes. A wide variety of cell surface peptide display systems are found in many microbial species and are well documented (Wang et al., 2021). *Escherichia coli* is a system in which various techniques of cell surface engineering are experimented with and confirmed. The metal-binding protein EC20, siderophore-binding protein, and CueR were individually examined for their ability to bind Pb, iron, and copper, respectively (Wang et al., 2021). The CadR gene from the wild-type *Pseudomonas putida* strain was modified in *Saccharomyces cerevisiae* and exhibited about a six-fold increase in the binding effectiveness for cadmium compared to the wild type strain (García-Hernández et al., 2017).

Additionally, Wei et al. (2014) changed *Escherichia coli* to make a strain that selectively takes Pb from solutions containing other heavy metals. They added the promoter section for PbrR and Pb-specific binding proteins. Similarly, Almaguer-Cantú et al. (2011) showed that incorporating a gene that expresses metallothionein from mice into *Escherichia coli* greatly improved the absorption of Pb-II.

Furthermore, Jafarian and Ghaffari (2017) found that adding a gene that codes for metallothionein (CgMT) to *Escherichia coli* BL21 (DE3) made it better at absorbing Pb ions, which helped get rid of Pb from polluted areas. Transgenic micro-organisms have benefits in the remediation of heavy metals, such as enhanced efficiency, resilience, and ecological preservation. However, their practical use is mainly restricted to laboratory trials. Before realizing widespread practical deployment, we need to conduct additional research to address safety, regulatory permission, and field application issues.

## 6 Research gaps and challenges

### 6.1 Mechanistic understanding at the molecular level

Although several methods of Pb resistance and remediation by microbes have been identified, there is still a dearth of full knowledge of the molecular processes involved. A comprehensive understanding of gene regulation, protein interactions, and metabolic alterations in response to Pb stress is crucial for improving and maximizing microbial remediation techniques. Furthermore, it is essential to conduct comprehensive genomic, transcriptomic, and proteomic research to discover and describe the genes and proteins that play a role in Pb detoxification. These studies may assist in developing micro-organisms that have improved capacities to remove Pb from the environment.

### 6.2 Optimization of microbial strain

The progress in creating genetically engineered or hybrid strains with enhanced capacities to remove Pb is still in the early stages. Further research is required to improve the efficiency and stability of these strains under various environmental situations. Further investigation is required to explore the possible advantages of using microbial consortia, consisting of numerous microbial species working together instead of a single strain, for Pb remediation. Gaining insight into the interactions among microbial consortia can enhance the efficacy of remediation efforts.

### 6.3 Interaction with native microbiota

The understanding of the interaction between imported Pb-remediating micro-organisms and native microbiota in contaminated environments is limited. Future research should investigate possible ecological disturbances and strategies to minimize adverse effects on the indigenous microbial populations. It is necessary to conduct long-term studies on the adaptability and development of microbial communities continuously exposed to Pb to comprehend microbial remediation’s long-term sustainability and dependability.

### 6.4 Environmental and operational factors

Further systematic research is required to determine the influence of several environmental parameters, such as pH, temperature, the presence of other heavy metals, and organic matter, on the effectiveness of microbial Pb remediation. Validating field and laboratory results is essential. Transferring successful experiments conducted in the laboratory to practical solutions that can be implemented on a broader scale in the field presents considerable difficulties. The research should prioritize the development of scalable procedures, including the design of bioreactors, conducting field experiments, and assessing the economic viability of large-scale operations.

### 6.5 Comparative studies with other remediation methods

There is a need for systematic comparative studies to evaluate the effectiveness of microbial remediation compared to standard physical and chemical techniques. Those studies should prioritize the evaluation of cost-effectiveness, environmental impact, and long-term sustainability to establish microbial remediation as a feasible option.---.

## 7 Conclusion and future perceptions

This review highlights the substantial potential of microbial remediation as a successful approach for reducing Pb contamination in agricultural soils and wastewater. Using bacteria, fungi, and microalgae makes it feasible to convert the toxic Pb into less harmful forms by employing processes such as biosorption, bioprecipitation, biomineralization, and bioaccumulation. The findings highlight the versatility and efficiency of microbial systems in immobilizing Pb, notably through the generation of nanoparticles that convert dissolved lead (Pb-II) into stable, less toxic states. Notably, *Pseudomonas* sp., *Bacillus* sp., and *Aspergillus niger* are among the commonly studied biosorbents for Pb-II removal. Exploring genetic engineering techniques and molecular-level mechanisms has provided a deeper understanding of how microbes develop resistance to Pb, revealing intricate detoxification pathways and resistance genes. Despite the promising advancements, challenges remain in the practical implementation of microbial remediation. Choosing the best temperature for EPS production and microbial growth is crucial, considering the appropriate microbial species and environmental conditions. The efficiency of removing Pb-II differs across various microbial species and is influenced by temperature changes. Temperature impacts microbial activity, including secretion content, biomass, and enzyme activities related to Pb-II binding. Addressing these challenges requires further research to develop robust, adaptable microbial consortia capable of thriving in diverse, contaminated environments. In conclusion, microbial remediation is promising for sustainable Pb detoxification in polluted environments. Continued interdisciplinary research and innovation are essential to overcome current limitations and fully realize the potential of microbial systems in restoring Pb-contaminated ecosystems.

## Author contributions

IG: Formal analysis, Software, Writing – review & editing. MA: Conceptualization, Formal analysis, Methodology, Project administration, Writing – original draft, Writing – review & editing. FL: Resources, Writing – review & editing. TL: Resources, Writing – review & editing. YC: Resources, Writing – review & editing. HL: Funding acquisition, Writing – review & editing. SL: Data curation, Methodology, Writing – review & editing. WF: Resources, Writing – review & editing.
